# The Effects of the Selective Removal of Adjacent Trees on the Diversity of Oak-Hosted Epiphytes and Tree-Related Microhabitats

**DOI:** 10.3390/plants13213019

**Published:** 2024-10-28

**Authors:** Agnese Anta Liepiņa, Diāna Jansone, Didzis Elferts, Ilze Barone, Jānis Donis, Āris Jansons

**Affiliations:** 1 Faculty of Medicine and Life Sciences, University of Latvia, Jelgavas Str. 3, LV-1004 Riga, Latvia; 2 Latvian State Forest Research Institute “Silava”, Rigas Str. 111, LV-2169 Salaspils, Latvia; diana.jansone@silava.lv (D.J.); didzis.elferts@silava.lv (D.E.); ilze.barone@silava.lv (I.B.); aris.jansons@silava.lv (Ā.J.)

**Keywords:** *Quercus robur* L., TreM, lichens, bryophytes, halo thinning

## Abstract

Restoration efforts to maintain oak-dominated habitats and enhance biodiversity often employ selective thinning, but its long-term effect on multiple taxa remains unclear. This study examines the effects of halo thinning around pedunculate oak on epiphytic and tree-related microhabitat (TreM) diversity in the boreal–nemoral forest zone. We revisited nine sites in Latvia with 150–331-year-old oaks, where thinning was conducted in 2003–2004. Epiphyte species composition and cover were assessed, and TreMs were evaluated using standardised methods. Diversity indices (Hill numbers, q = 0; 1; 2; 3) and statistical models (LMMs, Poisson GLMMs) were used to analyse the effects of thinning on species richness and TreM occurrence. Halo thinning over the past 20 years has not caused significant differences in epiphyte or TreM diversity, though managed trees exhibited a higher occurrence of the most common microhabitats. These findings suggest halo thinning may enhance specific TreM features but do not substantially promote epiphyte and TreM diversity. Future research should implement systematic monitoring, deriving the relationships between the conditions after the thinning and their effects, thus serving as the basis for adaptive habitat management strategies. Expanding the scope of such studies is essential for developing evidence-based forest management strategies.

## 1. Introduction

In the hemiboreal forests of Europe, pedunculate oak (*Quercus robur* L.) is a habitat for numerous bird, mammal, invertebrate, lichen, and moss species [1], suggesting a high potential for enhancing biodiversity in managed forests [2]. However, anticipated climatic changes are expected to reduce the vitality and productivity of oak trees [3,4,5]; therefore, specific management (e.g., thinning) has been implemented in an attempt to ensure a balance between the economic and biodiversity protection goals of the management [6].

Selective thinning is used in the boreal–nemoral forest zone to promote sustainable pedunculate oak growth and maintain the oak habitat structure [1,7]. One particular type of selective thinning is halo thinning. This method involves clearing trees in a circular formation around the canopy of a veteran tree, creating a “halo” effect [8]. Thinning may counteract secondary succession and enhance biodiversity in oak stands [9,10]. However, without comprehensive studies across multiple taxa and functional guilds, it remains uncertain whether biodiversity in these forests is better supported by allowing natural succession to progress without intervention or by restoring the previous semi-open stand structure [11].

Harvesting practices disturb microsite environmental conditions, potentially impacting the growth of understory vegetation [12]. Halo thinning affects light availability, soil temperature, moisture, and organic matter content, which may influence forest species’ spatial distribution [13]. By altering vital environmental factors, thinning affects the local microclimate, favouring some species and disadvantaging others, leading to shifts in specialised species composition [14,15]. Consequently, such modifications can directly or indirectly impact epiphytic species dispersal, establishment, and persistence [16].

The composition and abundance of epiphytic species in forests are influenced by environmental factors like light, moisture, and wind, with the species and size of host trees being essential [17]. Due to their physiological traits, epiphytes are effective indicators of forest continuity and management [18]. They are responsive to forest management and are strongly affected by a lack of structural diversity, responding more sensitively than vascular plants [19,20]. Structural differences in trees create environmental and resource gradients, leading to a significant diversity in epiphytic vegetation and distinct colonisation patterns [21].

Distinct and well-defined tree structures that provide specific locations for organisms to live, feed, or take shelter are known as tree-related microhabitats (TreMs) [22]. The presence of TreMs may indicate habitat naturalness and reflect forest ecological complexity. These microhabitats help evaluate and manage species richness across multiple taxa, serving as valuable biodiversity indicators [23]. Large oak trees host diverse TreMs and are recognised as biodiversity hotspots [22], providing valuable insights into ecosystem sustainability. Additionally, assessing the effects of forest management on biodiversity indicators can inform future oak management strategies for increased biodiversity in stands with retained oaks in the Baltic region.

Although creating openings around oak veteran trees is designed to improve oak growth and promote the associated biodiversity [8], its effects on epiphytes and TreMs remain contradicting. This study aimed to examine how the selective removal of adjacent trees around oaks affects the biodiversity of epiphytes and TreMs and to explore potential connections between these biodiversity indicators. We hypothesised that halo thinning would increase the diversity of the epiphytes and TreMs associated with the examined oaks.

## 2. Results

### 2.1. Tree and Stand Parameters

Between 2004 and 2023, several oak trees across multiple stands experienced mortality due to decay or treefall, and only the living trees were included in this analysis, from those 25 managed trees and 30 control trees. Overall, the oak trees had similar dimensions, with the mean circumference varying from 2.67 ± 0.74 m for control trees to 2.81 ± 0.86 m for managed oak trees (Figure S3). Furthermore, the mean height of managed and control trees was comparable, being 29.03 ± 4.68 and 29.25 ± 4.14 m, respectively. The control oak trees had a slightly greater mean crown area (120.88 ± 85.29 m²) than managed trees (173.15 ± 112.28 m²); however, these differences were not statistically significant (*p* = 0.06).

Local tree stand parameters (Figure S4) differed across both management types, with the mean stand basal area significantly higher in control plots (45.68 ± 13.30 m²) than in managed plots (34.96 ± 15.07 m²) (*p* = 0.01). Similarly, the managed plots had a significantly lower (*p* = 0.04) mean stand density than the control plots: 610.67 ± 233.16 and 412.80 ± 168.02 trees per ha, respectively. However, no significant differences were observed in the mean canopy layer tree density (*p* = 0.07), mean tree height (canopy layer (*p* = 0.40) and under-canopy layer (*p* = 0.61)), and mean spruce basal area (*p* = 0.27).

### 2.2. Epiphytes

**Figure 1:**
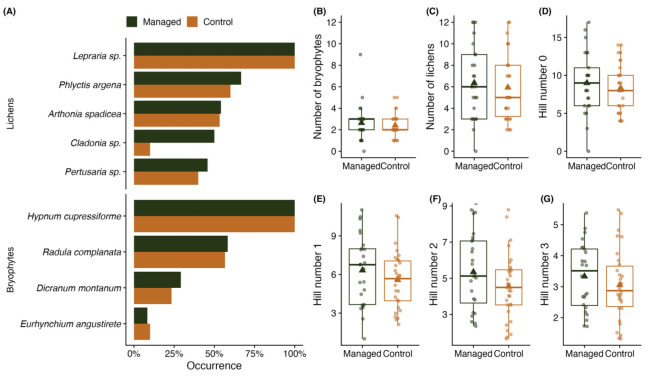
(**A**) Occurrence of most observed lichen and bryophyte species, (**B**) number of bryophyte species, (**C**) number of lichen species, (**D**) Hill number 0 of epiphytes, (**E**) Hill number 1 of epiphytes, (**F**) Hill number 2 of epiphytes, (**G**) Hill number 3 of epiphytes, by types of forest management. Triangle represents the mean value.

A total of 25 different bryophyte and 39 different lichen species were found on the studied oaks. Five of the detected bryophyte species (*Anomodon longifolius*, *Homalia trichomanoides*, *Isothecium alopecuroides*, *Metzgeria furcate*, *Neckera pennata*) were categorised as woodland key habitat indicator species [24]. Similarly, seven lichen species (*Acrocordia gemmate*, *Graphis scripta*, *Lecanactis abietina*, *Sclerophora* spp., *Arthonia byssacea*, *Arthonia spadicea*, *Arthonia vinosa*) belonged to this category; however, the latter four are also classified as protected species [25]. The most commonly found epiphyte species were *Hypnum cupressiforme* (present on 100% of the trees) and *Lepraria* spp. (100%) (Figure 1A,B). The woodland key habitat indicator species *Arthonia spacidea* was present on more than half of the trees, ranging from 53.3% to 54.2% on control and managed trees, respectively. The occurrence of the most frequently found epiphyte species on managed oaks was similar to control oaks, except for *Cladonia* spp., which was found on 50% of the managed trees and only 10% of the control trees.

The species richness and diversity of bryophytes and lichens were similar between the managed and control trees (Figure 1B–G). The mean Hill number 0 indicated that the managed trees had a slightly higher mean number of epiphytic species present (9 ± 4.04) than control trees (8.33 ± 3.10), but these differences were not statistically significant (*p* > 0.05). Furthermore, the total number of species was not affected by the circumference of the oak trees (not in general, nor when analysed amongst management types) (*p* > 0.05). The mean Hill number 1 of epiphytes ranged from 5.58 ± 2.21 for control trees to 6.33 ± 2.71 for managed trees (Figure 1E), with no statistically significant differences (*p* > 0.05), implying that the species evenness was comparable amongst managed and control trees. Similarly, the mean Hill number 2 was higher for managed oaks (5.35 ± 2.06) than for control oaks (4.54 ± 1.83) (Figure 1F), with no statistically significant differences (*p* > 0.05), signalling that the diversity of dominant species was similar for both tree groups. Likewise, the mean Hill number 3 ranged from 3.06 ± 1.14 for control trees to 3.33 ± 1.09 for managed trees, suggesting a similarly balanced distribution of dominant species (*p* > 0.05). Moreover, none of the local tree stand characteristics (total stand basal area, basal area of spruce, stand density, canopy tree height) influenced the Shannon–Wiener diversity index of epiphyte species (*p* > 0.05).

### 2.3. TreMs

**Figure 2:**
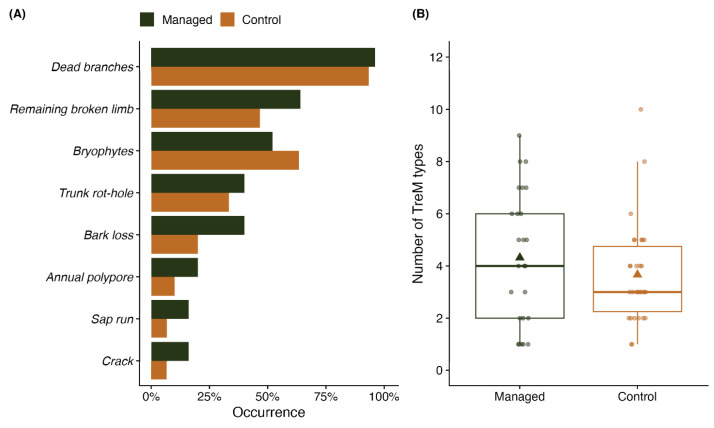
(**A**) Occurrence of TreM types, (**B**) number of TreMs per tree for managed and control oaks. Triangle represents the mean value.

The total number of TreMs identified per tree was similar between the two management types. The number of TreMs assessed increased with the tree diameter, but this effect was consistent across both management types and did not differ. No effect from other stand factors was observed on the number of TreMs evaluated. The average number of TreMs per tree was 4.3 ± 2.5 in managed stands, compared to 3.7 ± 1.9 in control stands (Figure 2B), with the difference being statistically insignificant (*p* > 0.05). Most TreMs observed on the trees were primarily associated with tree crowns, such as dead branches and broken limbs (Figure 2A). Dead branches were evenly present on trees from both management types. However, the “remaining broken limb” TreM was more commonly found on managed trees. This pattern was also observed for all the other most frequently detected TreMs, including trunk rot holes, bark loss, annual polypores, sap runs, and cracks, although “bryophytes” showed a different pattern, control trees having a higher number of this TreM.

**Figure 3:**
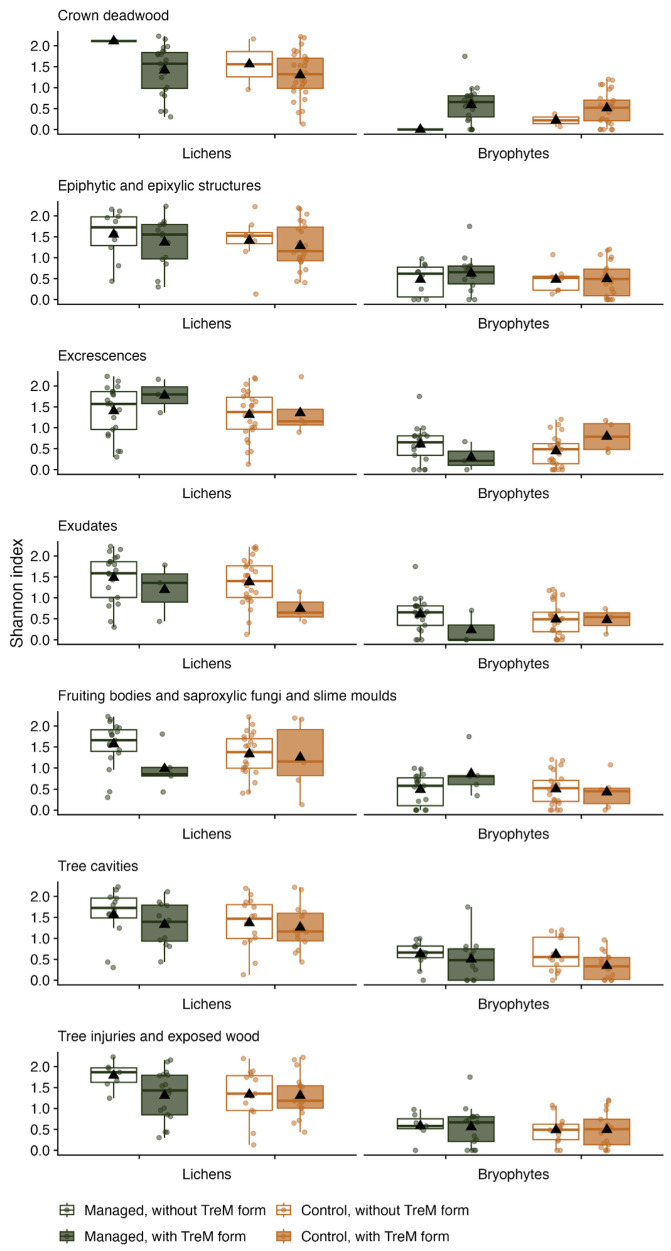
Shannon index values for bryophytes and lichens on trees exhibiting TreM forms by types of forest management. Triangle represents the mean value.

For the analysis of epiphyte diversity and TreM abundance interactions, the detected TreM types were divided into seven TreM forms: crown deadwood, epiphytic and epixylic structures, excrescences, exudates, fruiting bodies and saproxylic fungi and slime moulds, tree cavities, tree injuries, and exposed wood. The comparison of the bryophyte and lichen Shannon–Wiener diversity amongst oaks with and without the TreM forms showed somewhat similar tendencies (Figure 3), except for “crown deadwood”, which was found on nearly all of the trees (53 out of 55) and, therefore, was excluded from further interpretation. Oak trees without “exudates” had a significantly higher (*p* = 0.01) mean lichen diversity index (1.38 ± 0.54–1.48 ± 0.56) than trees with this TreM form (0.74 ± 0.37–1.19 ± 0.0.69), regardless of whether these were managed or control oaks. However, in the case of other TreM forms, no differences in lichen diversity were observed based on TreM form presence or absence on managed or control trees. Similarly, the presence/absence of detected TreM forms did not influence the bryophyte diversity on control or managed trees.

## 3. Discussion

Epiphytic species are known to be sensitive to forest management practices [19,20], and the selective removal of surrounding trees is often implemented to promote biodiversity in oak stands [9,10]. Based on this, we hypothesised that halo thinning would enhance the diversity of epiphytes and TreMs on the studied oaks. However, our results did not reveal significant differences in epiphytic diversity between managed and control trees, contradicting our hypothesis. All of the explored diversity proxies (Hill numbers 0–3) indicated that species richness and evenness, as well as the abundance and diversity of the dominant epiphytic species, were similar on managed and control oaks (Figure 1).

Furthermore, recent studies highlight that even low-intensity human disturbance can negatively affect epiphyte communities by reducing species richness and altering species composition [26]. Additionally, partial thinning has been shown to harm epiphytic bryophytes due to increased wind exposure, light penetration, and temperatures, all of which reduce forest moisture [27]. Similarly, some experiments report a negative impact from partial cutting for epiphytic lichens, largely due to the same environmental changes [28]. In contradiction, other studies from the Baltics have reported crown openings positively affecting lichen species abundance [29]. This partially aligns with our findings, as we observed that the occurrence of *Cladonia* spp. lichen species was more than twice as high on managed oaks as those without halo thinning around them (Figure 1A). Nonetheless, while the average number of epiphytic species on managed trees was not significantly higher than on control trees, the mean species richness was not reduced either. This implies that, although thinning created noticeably different stand conditions between the studied plots (see Section 4.1), the intensity of thinning may have been mild enough to avoid significant changes in the microclimate, thus mitigating major effects on epiphyte diversity.

Oak trees are recognised as biodiversity hotspots due to their ability to support a wide range of TreMs [22]. The geographic location and microclimate of the region influence the diversity of TreMs [30]. Additionally, the formation of TreMs differs significantly between unmanaged and managed forests, with unmanaged forests exhibiting higher rates of features such as rot holes, polypores, exposed heartwood, burr cankers, and bark loss [31,32]. In Poland, TreMs associated with pedunculate oak [33] were predominantly related to bark characteristics, including insect galleries and bark pockets, along with epiphytic cryptogams and crown deadwood, which were also the most common TreM types observed in our study (Figure 2). Moreover, in this study, all of the most frequently detected TreMs, except for the “bryophytes” type, were more prevalent on managed trees, adding to the contradiction of our hypothesis. This exception is likely because bryophytes, as a dynamic TreM type, are particularly sensitive to microclimatic changes and less resilient to disturbances [17].

The presence of TreMs and tree-specific characteristics, such as tree size [34], bark rugosity, and crown leaf density [35], significantly shape epiphyte distribution [13]. While evidence links forest biodiversity to TreM abundance at the stand scale, the relationship with direct biodiversity indicators tends to be moderate [36,37]. Similarly, our study found limited evidence of distinct interactions between specific TreM forms, forest management, and epiphytic diversity (Figure 3). However, a notable pattern emerged for the “exudates” TreM form, where oak trees without exudates exhibited a higher lichen diversity across both managed and control trees. This may be attributed to changes in bark properties, as bark texture, chemistry, and moisture retention are key factors influencing lichen communities on seed-producing trees [38]. However, research on the impact of bark exudates on lichens regarding *Quercus* spp. species is limited, and we speculate that the chemical components of these exudates could alter the bark surface, making it less habitable for common lichen species. Moreover, the height at which exudates were assessed corresponds to the height used for lichen evaluation. This may explain the lack of correlation with other microhabitat types, such as those associated with the canopy.

In conclusion, the selective removal of neighbouring trees around large oaks over the past 20 years has not led to significant differences in epiphyte diversity nor promoted the expected increase in epiphyte and TreM diversity. However, the most common microhabitats were more frequently observed on managed trees, indicating that small-scale thinning may enhance the presence of microhabitats on these relict oak trees in the Baltic region. Lichen diversity was negatively affected by stem exudates, although no other notable interactions between specific TreM forms and epiphyte diversity were identified. The structural differentiation between tree zones, such as the trunk and crown [39], along with variations in light and humidity along the forest’s vertical gradient [40], likely shapes epiphyte communities. The current study characterised the epiphytic diversity on the lower part of the oak stems (1.3 m), which may not fully represent typical epiphyte distribution patterns across the entire tree. Sampling at 1.3 m limits the conclusions about epiphyte diversity on the oaks overall, as epiphytes may exhibit different community structures in higher stem or crown zones due to varying microclimatic conditions [41]. Future research should therefore expand the focus of selective tree removal studies to include the upper parts of tree stems and branches, as epiphyte species may shift their vertical distribution over time in response to specific microclimatic conditions [41]. Additionally, future research should implement systematic monitoring, deriving the relationships between the conditions after the thinning and their effects, thus serving as the basis for adaptive habitat management strategies.

## 4. Materials and Methods

### 4.1. Description of the Study Sites

**Figure 4:**
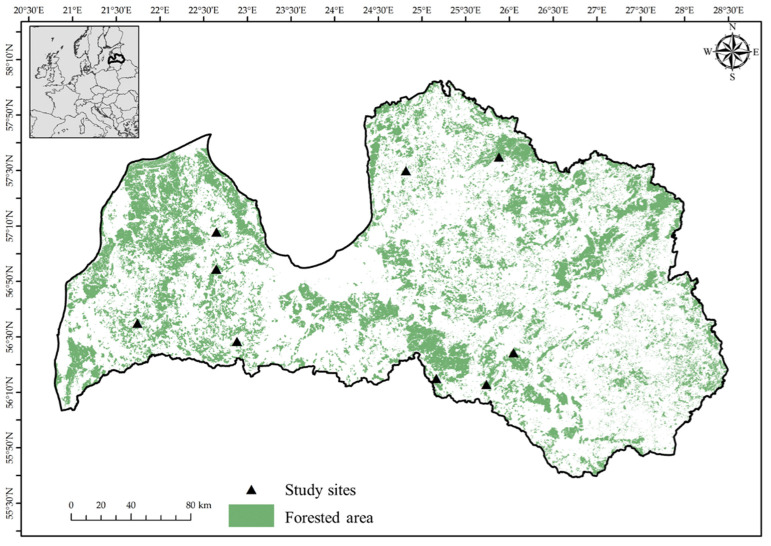
Locations of the study sites (black triangles), with the forest areas represented in green.

For this study, we revisited nine previously established forest study sites located in the central and western parts of Latvia (Figure 4), where the mean yearly temperature for the last 30 years has been 6.8 °C, and the annual precipitation has reached 685.6 mm [42]. The selected stands represented a comprehensive range of soil conditions, from oligotrophic to eutrophic, including dry, wet, and drained forest types (*Oxalidosa*, *Myrtillosopolytrichosa*, *Hylocomiosa*, *Aegopodiosa*, *Myrtillososphagnosa*, *Myrtillosopolytrichosa*). Furthermore, the stands were primarily composed of silver birch (*Betula pendul*a), Norway spruce (*Picea abies*), and aspen (*Populus tremula*), along with pedunculate oak and Scots pine (*Pinus sylvestris*). The stand ages ranged between 17 and 205 years, with an average of 94 ± 44 years (mean ± standard deviation), and the size of the forest stands ranged between 1.2 and 7.8 ha, an average of 2.9 ± 2.3 ha.

Despite the different stand conditions, these forest areas were initially selected because they all featured old, large oak trees aged between 150 and 331 years, averaging 230.3 ± 22.3 years. Moreover, based on their branch disposition, these oak trees were remnants from earlier wooded meadows, persisting as relics within the current forest structure. In 2003 and 2004, halo thinning was implemented around oak trees under the guidance of experts from the State Forest Service. Oak trees grew scattered across the forest stands, and thinning was performed around four individual oaks within each stand (hereafter referred to as managed oaks). Although the exact volume of wood and stand basal area removed during the thinning was not documented, based on observations in the forest, thinning occurred within a 10 m radius around each oak tree (Figure S1). Additionally, four other oaks with comparable dimensions, positioned at a moderate distance from the managed trees, were selected and classified as control trees within each stand. In 2023, data were collected in the same forest stands to evaluate the effects of halo thinning around large oak trees on the diversity of epiphytic species and TreMs.

### 4.2. Data Collection and Measurements

For each studied oak tree, measurements were made of the circumference (m) at breast height (H = 1.3 m), tree height (m), and crown width (m) along the north–south (N-S) and east–west (E-W) axes. Furthermore, a circular survey plot (radius = 12.62 m, 500 m²) was established at 1.41 m to the southeast from the stem. All standing trees (diameter at breast height (DBH) > 6.1 cm) were measured within the sample plot area. Data on tree species, height, and diameter were collected, along with an assessment of the canopy layer to which each tree belonged.

For the TreM evaluation, a standardised detection method from [22] was applied to ensure data consistency. This methodology categorises seven forms of TreMs based on their morphology and importance to biodiversity, including cavities, tree injuries, crown deadwood, excrescences, fruiting bodies, epiphytic and epixylic structures, and exudates. These are further classified into 15 groups and 47 specific types in a hierarchical system. The assessment of TreMs took place during the leafless period in the autumn of 2023.

The composition and cover of each bryophyte and lichen species on the tree trunks were described in four plots on each of the surveyed living oaks. The plots were divided based on two parameters, cardinal direction (north, west, south, east) and height from the tree base; in each cardinal direction, a 10x50 cm plot (frame) was installed, divided into five 10x10 cm large subplots (Figure S2). The shorter side of the frame (10 cm) was attached horizontally to the tree at a height of 1.3 m, and the direction of the longer side was directed downwards. If the identification of the bryophyte or lichen species was not possible in the field, samples of the species were collected for further identification in the laboratory. Lichen species nomenclature follows [43], and bryophyte species nomenclature follows [44].

### 4.3. Data Analysis

The mean values of stem circumference, tree height, and crown area were calculated to assess oak dimensions depending on management status. The forest stand characteristics for each plot were evaluated by calculating the total stand basal area, basal area of spruce, canopy layer density, and total stand density. In addition, average values for canopy tree height and understorey tree height were determined for each plot. For the comparison between managed and control oak trees, linear mixed-effects models (LMMs) were applied, utilising the R packages “lme4” [45] and “lmerTest” [46]. Separate models were constructed for each parameter of interest. In all models, the name of the study site location was included as a random effect to account for potential intra-site correlation, as multiple oaks originated from the same forest stand.

To evaluate the effects of halo thinning on epiphyte species diversity 20 years post-management, the mean relative epiphyte cover of the 20 stem subplots for each tree was calculated. The number of epiphyte species on each tree was then determined and categorised into bryophytes and lichens. Subsequently, the Hill numbers with the different q order (q = 0, 1, 2, 3) [47] as implemented in R package diverse [48] were calculated for each tree. The index values between management types were compared using LMMs. Similarly, the LMMs were used to assess whether the differences in local stand parameters significantly influenced epiphyte diversity. Furthermore, the number of TreM types was estimated for all trees, and the distribution of the most prevalent TreMs and epiphyte species on managed versus control trees was characterised. Concurrently, the number of lichen species, bryophyte species, total species, and TreMs amongst managed and control trees were compared using Poisson generalised linear mixed-effects models (GLMMs) [45].

Lastly, to detect potential interactions between the presence of TreMs and bryophyte and lichen diversity, TreM types were classified according to their respective forms [22]. Epiphyte diversity was analysed based on whether halo thinning was applied around the oak and whether a particular TreM form was present on the tree. The influence of TreM forms and management interaction on diversity indexes was analysed using LMMs (a separate LMM for each TreM form). All statistical analyses were performed in the program R version 4.4.1. [49].

## Data Availability

Dataset available on request from the authors.

## Supplementary Materials

The following supporting information can be downloaded at: https://www.mdpi.com/article/10.3390/plants13213019/s1, Figure S1: Halo thinning around a veteran oak tree at a studied forest stand; Figure S2: Schematic picture of epiphyte assessment plots; Figure S3: (A) Height, (B) circumference and (C) tree crown projective cover by types of forest management; Figure S4: Parameters characterising the stand by types of forest management: (A) total basal area, (B) Norway spruce basal area, (C) tree density, (D) first floor (canopy layer) tree density, (E) first floor mean tree height, (F) second floor (under-canopy layer) mean tree height.
