# Supplementary figures and images for: The Effects of the Selective Removal of Adjacent Trees on the Diversity of Oak-Hosted Epiphytes and Tree-Related Microhabitats
Source: Plants (Basel). 2024 Oct 28;13(21):3019. doi: 10.3390/plants13213019 (PMC11548538; doi:10.3390/plants13213019)

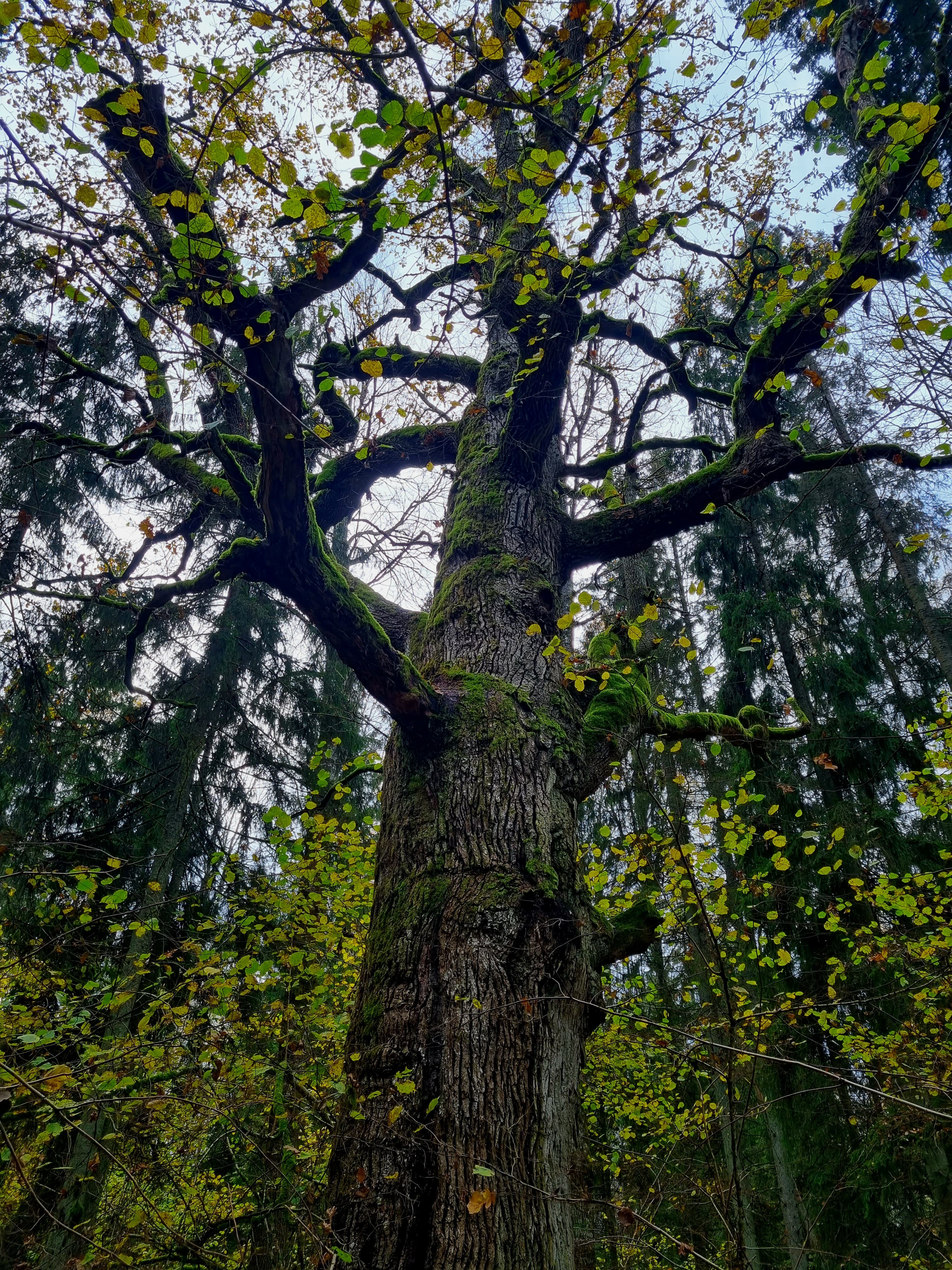

Supplement: Supplementary file 1 [file plants-13-03019-s001.zip › Figure_S1.jpeg]

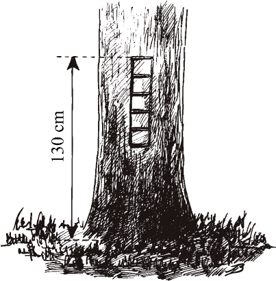

Supplement: Supplementary file 1 [file plants-13-03019-s001.zip › Figure_S2.png]

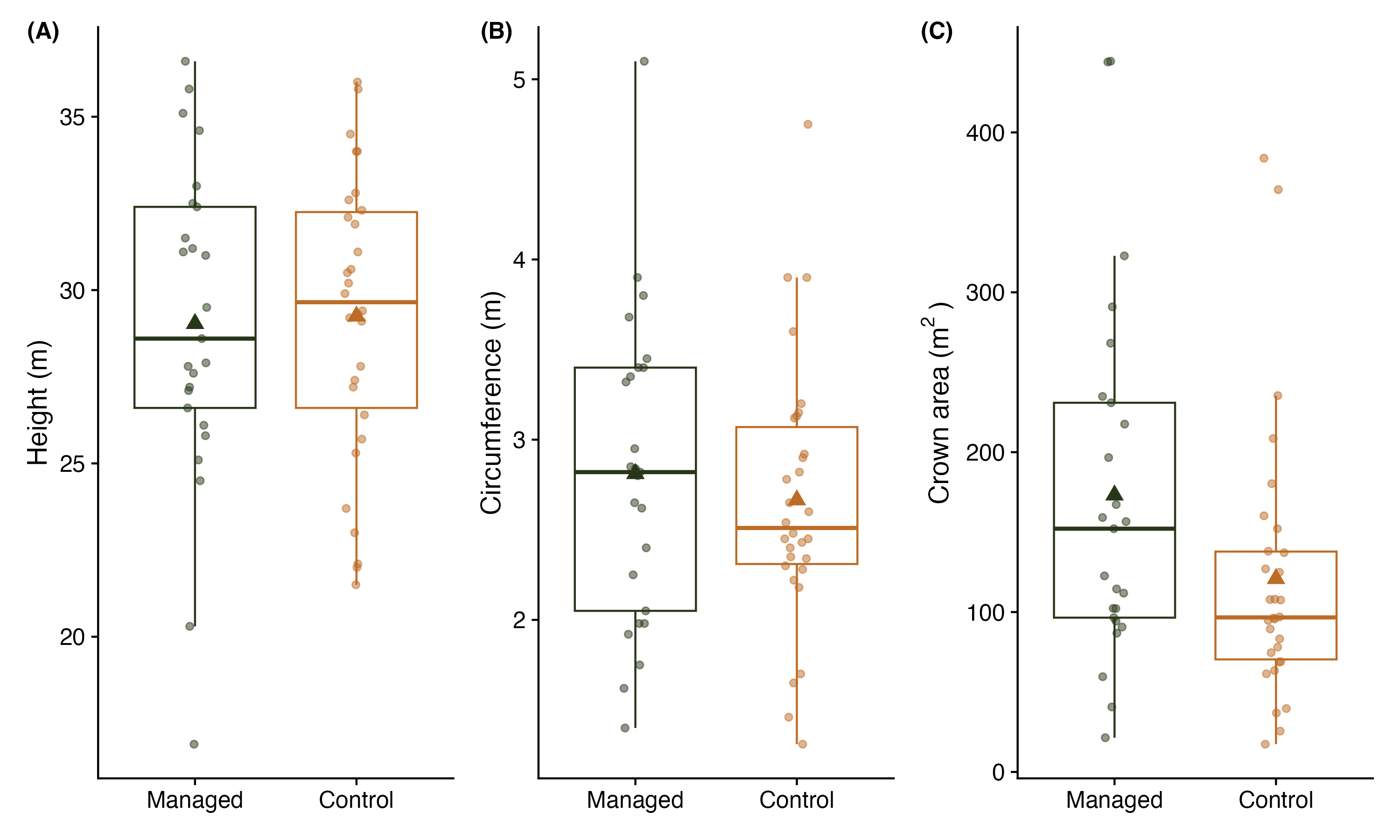

Supplement: Supplementary file 1 [file plants-13-03019-s001.zip › Figure_S3.png]

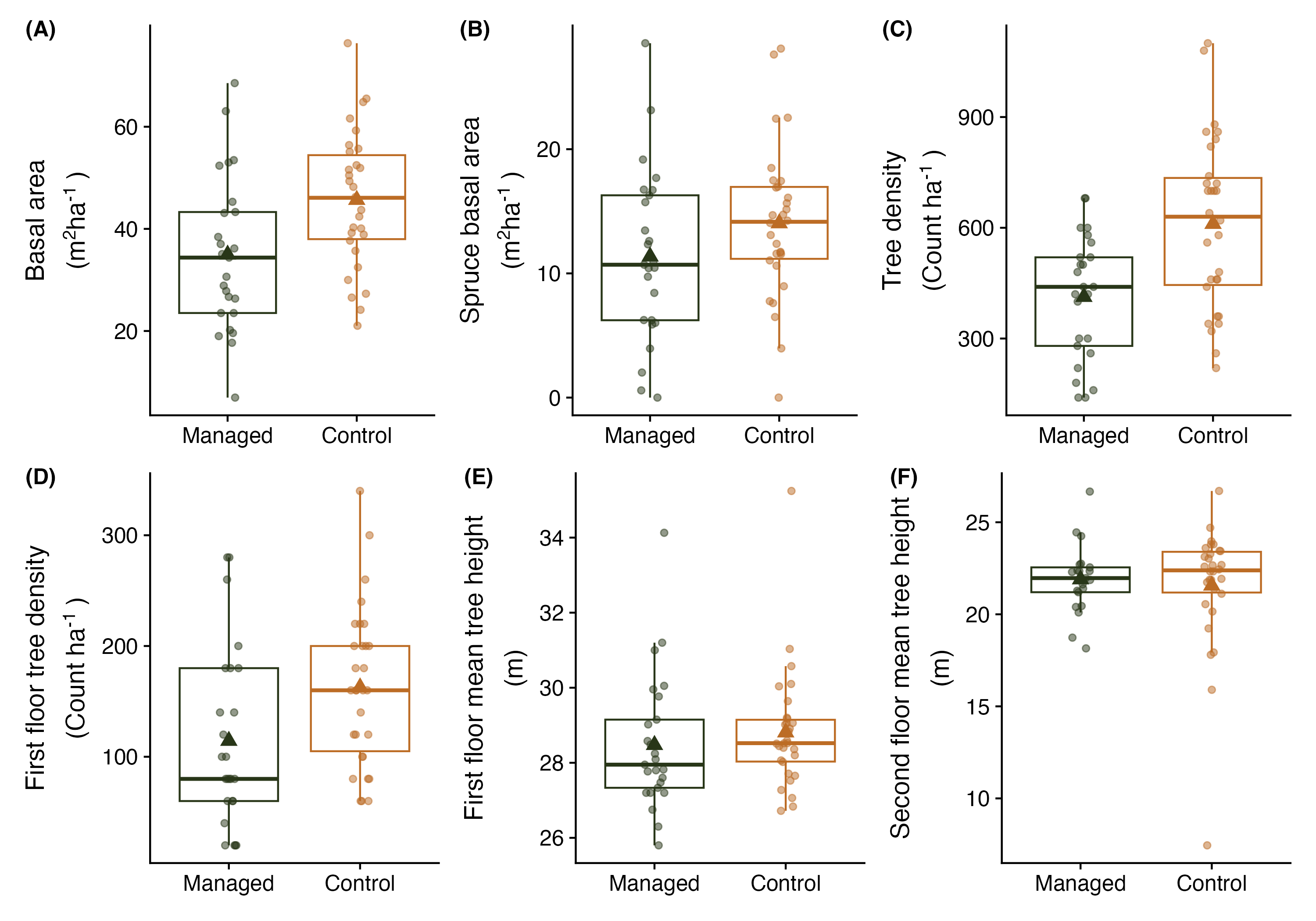

Supplement: Supplementary file 1 [file plants-13-03019-s001.zip › Figure_S4.png]
